# Primary Hepatic Squamous Cell Carcinoma: A Case Report

**DOI:** 10.7759/cureus.63803

**Published:** 2024-07-04

**Authors:** Manal Lyagoubi, Chourouq Mehdaoui, Anass Haloui, Nassira Karish, Zahi Ismaili, Amal Bennani

**Affiliations:** 1 Pathology, Faculty of Medicine and Pharmacy of Oujda, Mohammed 1st University, Oujda, MAR; 2 Pathology, Mohammed VI University Hospital, Faculty of Medicine, Mohammed 1st University, Oujda, MAR; 3 Gastroenterology and Hepatology, Mohammed VI University Hospital, Faculty of Medicine, Mohammed 1st University, Oujda, MAR; 4 Hepato-Gastroenterology, Mohammed VI University Hospital, Faculty of Medicine, Mohammed 1st University, Oujda, MAR; 5 Anatomopathology, Faculty of Medicine and Pharmacy of Oujda, Mohammed 1st University, Oujda, MAR

**Keywords:** immunotherapy, malignancy, case report, liver, scc, keratinizing squamous cell carcinoma, squamous cell carcinoma of unknown primary

## Abstract

Primary squamous cell carcinoma (SCC) of the liver, a notably uncommon type of cancer, is frequently linked with diverse hepatic conditions including hepatic cysts, hepatolithiasis, and hepatic teratoma. Literature indicates that only approximately 30 cases of primary SCC of the liver have been documented. Herein, we report a 54-year-old previously healthy patient who was presented with cholangitis symptoms. Examinations revealed normal vitals. However, deranged liver function with transaminitis and hyperbilirubinemia were noticed. A CT scan showed a hepatic mass with bile duct dilation. Biopsy confirmed hepatic squamous cell carcinoma, leading to chemotherapy treatment. Despite treatment, the survival outcomes for this cancer remain limited, and the prognosis is generally unfavorable.

## Introduction

Squamous cell carcinoma (SCC), a type of cancer that develops in the squamous cells, commonly originates through the malignant transformation of squamous cells located in organs lined with squamous epithelium, including the skin, distal esophagus, urinary tract, lungs, cervix, and rectum [1]. This type of malignancy represents 4% to 5% of cancers with unknown primary sites [2]. In the liver, SCCs are predominantly metastatic, originating from primary sites like the lung, thyroid, or gastrointestinal tract [1]. Primary SCC of the liver is exceptionally rare, with only 33 cases documented in the English literature [3]. Research has indicated a link between primary SCC of the liver and factors such as male sex [4], hepatic cysts, chronic cholecystitis, hepatolithiasis, or hepatic teratoma [1]. The pathogenesis of this condition remains unclear, although chronic inflammation or irritation of the bile duct or congenital cysts are believed to encourage secondary squamous metaplasia and subsequent transformation [1]. Systemic chemotherapy and surgical intervention have the potential to achieve full remission in cases of poorly differentiated squamous cell carcinoma (SCC) of the liver. Additionally, there is evidence of positive outcomes with hepatic arterial injections of low-dose chemotherapeutic agents [3,5]. This rare and aggressive cancer has a dismal prognosis, with previous studies indicating an overall survival of less than 12 months [1].

## Case presentation

**Figure 1:**
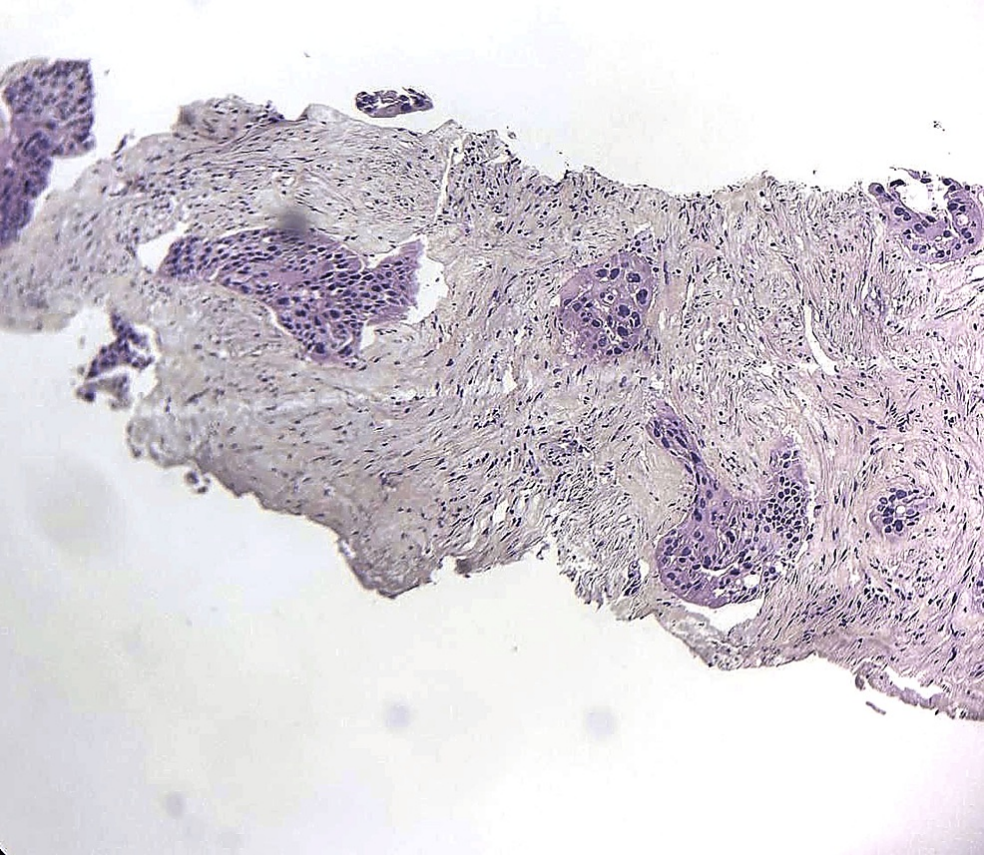
Microphotography revealing the carcinomatous proliferation made of large polygonal cells with abundant eosinophilic cytoplasm. (HE, 200X)

**Figure 2:**
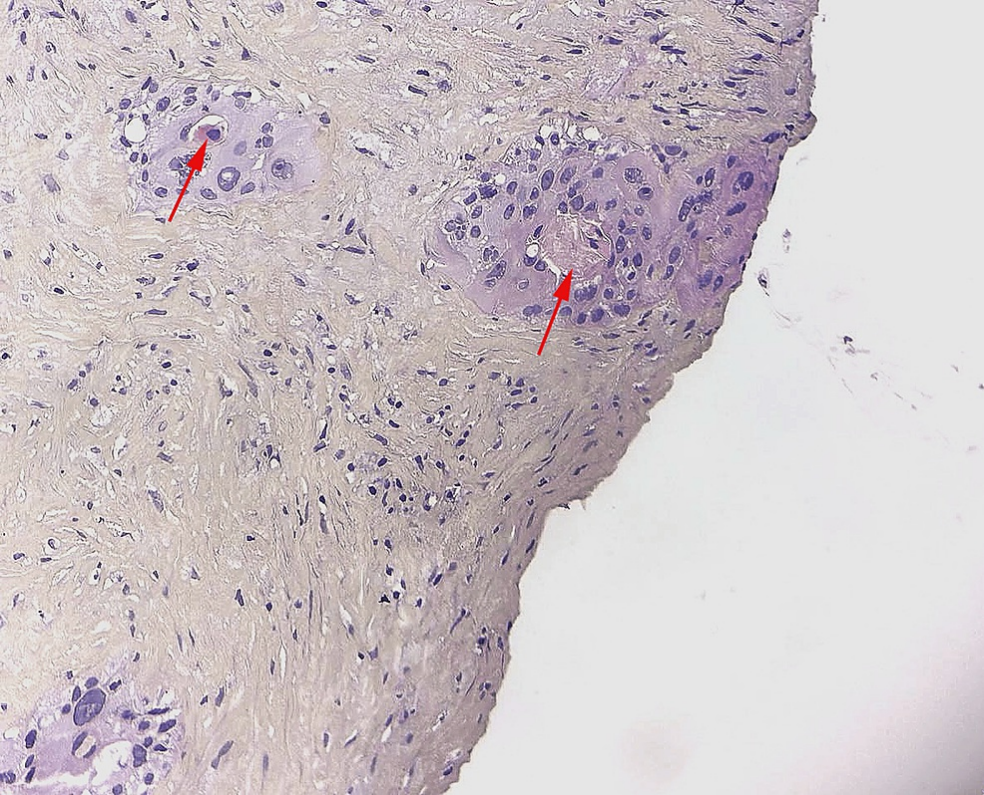
The tumor cells showed keratosic maturation in some clusters forming structures resembling horn pearls (Red arrows). (HE, 400X)

A 54-year-old patient with no notable medical history presented with symptoms of cholangitis characterized by a fever, jaundice, and abdominal pain in the right hypochondrial region. Physical examination at admission showed a normal conscious patient. Vital signs showed a body temperature of 39.4°C, a blood pressure of 130/80 mmHg, a pulse rate of 95 bpm, and a respiratory rate of 15 breaths/min. The abdominal examination revealed splenomegaly and abdominal tenderness upon palpation of the right iliac fossa. The dermatological examination was unremarkable with no skin lesions suggestive of malignancy. The Ear, Nose, and Throat (ENT) examination with nasofibroscopy found no lesions in the ENT area. The biological examination revealed elevated total bilirubin at 5 mg/dL, elevated conjugated bilirubin at 4 mg/dL, and high gamma-GT and elevated alkaline phosphatases. Tumor markers including cancer antigen (CA 19-9), alpha-fetoprotein (AFP) and carcinoembryonic antigen (CEA) showed normal values. A contrast CT abdominopelvic revealed the presence of a solid tissue mass occupying segments IV and II and measuring 95 x 79 mm. This mass was associated with dilation of the left intrahepatic bile duct. The CT also revealed splenomegaly and minimal pelvic ascites. Vascular involvement was also identified. A CT scan-guided biopsy of the identified hepatic lesion was performed. Histopathological examination revealed hepatic parenchyma infiltrated by a carcinomatous proliferation of large polygonal cells with abundant eosinophilic cytoplasm and hyperchromatic atypical nuclei often in mitosis. The tumor cells showed keratosis maturation in some clusters forming structures resembling horn pearls (Figures 1, 2).

**Figure 3:**
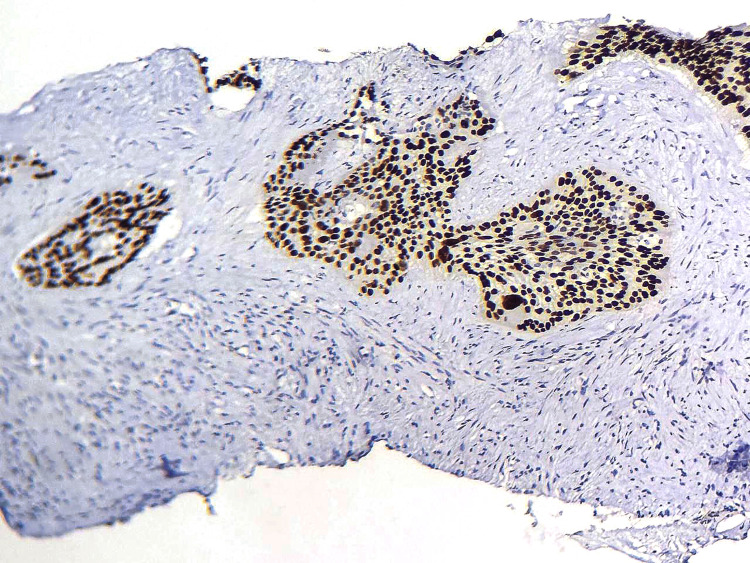
Immunohistochemical study revealed expression of P63 by the tumor cells. (HE, 400X)

**Figure 4:**
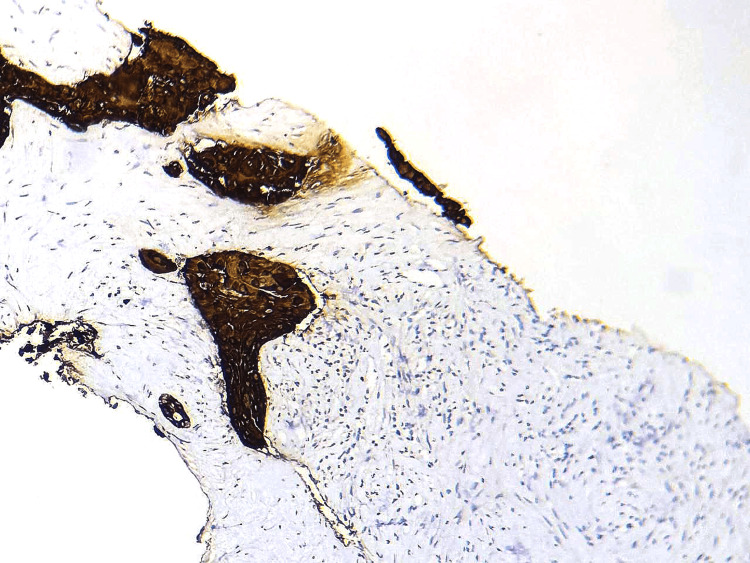
Immunohistochemical study revealed expression of CK5/6 by the tumor cells. (HE, 200X)

An immunohistochemical study revealed the expression of P63 and CK5/6 by the tumor cells, indicating squamous differentiation. The diagnosis of hepatic squamous cell carcinoma was made (Figures 3, 4).

As part of the extension assessment, colonoscopy and esophagogastroduodenoscopy were performed. Both returned normal. The thoracic CT did not reveal any lesions or pulmonary nodules.

Therapeutically, surgery was not performed due to locally advanced disease with vascular involvement. After a multidisciplinary discussion, the patient was referred for chemotherapy based on 5-fluorouracil.

The patient is currently followed up in consultation with a reduction in tumor size on the control scan after a three-month follow-up. The follow-up CT scan revealed a mass measuring 75 x 51 mm. A CT scan will be performed every six months to ensure follow-up of the patient.

## Discussion

Primary SCC (squamous cell carcinoma) of the liver is an exceedingly rare condition, with a very low incidence rate, with just 33 cases documented in the English literature from the 1970s to the present [3].

The exact underlying mechanism of this disease remains uncertain. Nonetheless, it is hypothesized that ongoing irritation from chronic inflammation of the biliary duct epithelium and/or congenital liver cysts may lead to squamous metaplasia and subsequent malignant transformation [1,6,7].

Data supporting this includes reports of a likely link between primary SCC and pre-existing or concurrent conditions such as liver cysts and hepatolithiasis in the majority of cases [3].

Many studies indicate a correlation between primary SCC and benign hepatic cysts, but the exact carcinogenic process has not been conclusively identified. It is hypothesized in several publications that the lining of benign cysts may undergo a dysplasia-metaplasia sequence that eventually leads to malignant SCC [8].

There are also a few instances where a potential association with hepatic teratoma was reported [9].

In our case, the patient was diagnosed with keratinizing SCC of the liver with no evident associated lesion.

Previous research indicates that the clinical presentation of primary SCC of the liver is typically vague. Patients often experience nonspecific symptoms such as blunt abdominal pain or discomfort, jaundice, unintended weight loss, and a palpable mass in the right upper quadrant with tenderness [1, 4]. On physical examination, a palpable abdominal mass and tenderness in the right upper quadrant, often accompanied by fever, can be identified [4]. Our patient exhibited fever pain and jaundice.

Unlike hepatocellular carcinoma, there are no specific serum markers for primary SCC of the liver [1]. Common laboratory findings include elevated levels of aspartate transaminase (AST), alanine transaminase (ALT), and bilirubin, which are likely attributable to chronic inflammation in the bile ducts or liver cysts, as well as tumor invasion [3]. It is also possible for patients to present with normal liver enzyme levels [1].

Regarding imaging techniques, CT scans are pivotal for preoperative assessment [3]. Typical findings often show a mass of slightly low density. During the arterial phase, the imaging may display uneven or mild enhancement or marginal enhancement, followed by enhancement in the portal and delayed phases. Some patients also present with intrahepatic bile duct stones, dilatation of the intrahepatic bile duct, and hepatic cysts [6]. Diagnosis can be challenging when the CT scan does not clearly reveal a liver mass but shows only liver cysts or hepatolithiasis before surgery [3]. In our patient's situation, CT imaging revealed a solid tissue mass occupying segments IV and II and measuring 95 x 79 mm.

Primary SCC of the liver is generally identified through a process of elimination, as metastatic SCC is more likely to occur in this organ [10]. Therefore, it is crucial to rule out primary sources such as the skin, head, neck, and lungs [3]. In our patient's case, laryngoscopy revealed no primary sources of the SCC.

A liver biopsy is the definitive test for confirming the diagnosis. Immunohistochemical (IHC) staining typically shows positivity for CK5/6, p63, and p40 [1]. A positive result for CK19 indicates the tumor cells originate from bile ductules, while positivity for CK7, 8, 14, or 5/6 suggests a keratinized squamous epithelial origin [3]. Reports also indicate some tumors exhibiting PDL-1 positivity [1].

In contrast, immunohistochemical staining for hepatocellular carcinoma would typically be positive for arginase 1, gpc3 hepatocytes, and AFP, and for cholangiocellular carcinoma, it would be positive for CK19 and CK8 [1]. The biopsy of our patient confirmed the diagnosis, showing strong, diffusely positive p63 immunostaining. Additionally, the FoundationOne CDx test was conducted to pinpoint any specific molecular changes in the patient’s cancer that could align with targeted therapies and to evaluate microsatellite instability and tumor mutational burden for potential benefits from immunotherapy [11].

Various treatments have been employed for primary SCC of the liver, including surgical resection, chemotherapy, radiotherapy, and transcatheter arterial chemoembolization. Early surgical intervention, before the tumor spreads to the surrounding liver parenchyma, is associated with a favorable prognosis [5, 12, 13]. Weimann et al. reported a survival of over four years following surgical resection alone, without any adjuvant chemotherapy or radiation therapy [5].

Surgical resectability and operability are critical factors that significantly influence prognosis [14].

In many cases, primary SCC of the liver is diagnosed at an advanced stage. In such instances, combining radiation therapy or chemotherapy with agents like 5-FU and/or Cisplatin with surgery is advisable [13]. Boscolo et al. reported successful treatment of advanced primary SCC with systemic therapy using CDDP and 5-FU along with surgical resection [15].

The clinical progression of primary SCC of the liver is typically aggressive [3]. The prognosis is extremely poor, typically resulting in survival of less than one year because the tumor is often detected in its advanced stages [16]. There have been instances of complete remission in cases of poorly differentiated SCC following systemic chemotherapy (cisplatin and 5-fluorouracil) combined with surgery [4, 10]. In cases where tumor recurrence is identified at a late stage, options like reoperation, systemic chemotherapy, or hepatic artery infusion chemotherapy are considered [3].

## Conclusions

Primary liver squamous cell carcinoma (SCC) is an exceedingly uncommon occurrence. It is believed to correlate with diverse hepatic conditions. Typically, the clinical manifestation lacks specificity, and there is still no particular serum indicator available. Herein, we report a 54-year-old previously healthy patient who presented with cholangitis symptoms. A hepatic mass with bile duct dilation was detected on a CT scan. Subsequent biopsy confirmed the presence of hepatic squamous cell carcinoma, following examinations that showed normal vitals but elevated bilirubin and liver enzymes. This diagnosis led to the decision to begin chemotherapy treatment. A standardized treatment regimen for this disease has not yet been defined; nevertheless, available therapeutic measures vary from surgical intervention to palliative approaches, contingent upon individual cases.
